# Pyrotinib with trastuzumab and aromatase inhibitors as first-line treatment for HER2 positive and hormone receptor positive metastatic or locally advanced breast cancer: study protocol of a randomized controlled trial

**DOI:** 10.1186/s12885-020-07143-2

**Published:** 2020-07-13

**Authors:** Changjun Wang, Yan Lin, Yidong Zhou, Feng Mao, Hanjiang Zhu, Jinghong Guan, Xiaohui Zhang, Songjie Shen, Xin Huang, Chang Chen, Ru Yao, Jialin Zhao, Qiang Sun

**Affiliations:** 1 Department of Breast Surgery, Peking Union Medical College Hospital, No.1 Shuaifuyuan, Dongcheng District, Beijing, 100730 China; 2 Department of Dermatology, 90 Medical Center Way, Surge 110, University of California, San Francisco, CA 94143-0989 USA

**Keywords:** Metastatic breast cancer, Pyrotinib, Aromatase inhibitors, Trastuzumab

## Abstract

**Background:**

HER2 dual-blockade combined with aromatase inhibitors (AI) is a promising strategy to improve progression-free survival (PFS) in hormone receptor (HR) positive, metastatic breast cancer (MBC). Pyrotinib is a novel irreversible epidermal growth factor receptor/HER2 dual tyrosine kinase inhibitor. However, there is scarcity of data on the effectiveness and safety of pyrotinib combined with trastuzumab and AI as first-line treatment in a metastatic setting.

**Methods/design:**

The present study is a prospective, randomized, open-label trial. 198 patients with HER2+/HR+ MBC will be recruited. Eligible patients will be allocated (2:1) to either an experimental group (pyrotinib + trastuzumab + AI) or a control group (trastuzumab + AI). Allocation will be stratified by 1) time since adjuvant hormone therapy (≤ 12 months/> 12 months/no prior hormone therapy); 2) lesion sites (visceral / non-visceral). The primary endpoint is PFS.

**Discussion:**

To our knowledge, this is the first prospective randomized controlled trial to assess dual HER2-blockade with pyrotinib in the metastatic setting. This study will provide valuable evidence regarding the efficacy and safety of pyrotinib when combined with trastuzumab and an AI as first-line treatment for MBC. Moreover, it will also evaluate the feasibility of endocrine therapy as an alternative to chemotherapy in providing de-escalation therapy with less toxicity for advanced HR+/HER2+ patients.

**Trial registration:**

ClinicalTrials.gov, ID: NCT03910712. Registered on 10 Apr. 2019.

## Background

The *Her-2/neu* gene was first discovered in 1987 and acts as a prognostic indicator of poor survival of breast cancer (BC) [1]. Trastuzumab, a HER2-targeting monoclonal antibody, improves survival for HER2+ BC owing to its prominent anti-HER2 effect [2]. Given that nearly half of HER2+ metastatic breast cancer (MBC) presents with hormone receptor (HR) positivity, several studies have revealed that HER2 and estrogen receptor (ER) signaling pathways have widespread crosstalk [3–6]. The ER signal pathway acts as an escape mechanism to enable the cancer cell to bypass HER2 blockade signal transduction and facilitate carcinogenesis and progression [7]. Therefore, hypothetically, the combination of anti-HER2 treatment and endocrine therapy (ET) could serve as a more effective method to treat HER2+/HR+ MBC.

Several trials have evaluated the efficacy of aromatase inhibitors (AI) together with single anti-HER2 agents (trastuzumab or lapatinib) to treat HER2+/HR+ MBC. They demonstrated a prolonged progression-free survival (PFS) ranging from 4.8–14.3 /months [8–10] (Table 1). Novel anti-HER2 agents (such as pyrotinib and pertuzumab) have also provided promising strategies, since dual HER2-blockade has been found to be effective in both the metastatic and neoadjuvant settings [13–15].

**Table 1:** Summary of previous studies of first line ET+ anti-HER2 treatment for MBC

Trial	Regimen	Size	Median PFS/TTP (months)			Patinets
			Control	Experiment	hazard ratio (95% CI)	
Kaufmann et al.(TAnDEM) [8]	Anastrozole+/−trastuzumab	207	2.4	4.8	0.63(0.47–0.84)	First line
Johnston et al. (EGF30008) [10]	Letrozole+/−lapatinib	219	3	8.2	0.71(0.53–0.96)	First line
Huober et al. (eLEcTRA) [9]	Letrozole+/−trastuzumab	92	3.3	14.1	0.67(0.35–1.29)	First line
Johnston et al. (ALTERNATIVE) [11]	AI+/−trastuzumab +/−lapatinib	355	8.3/5.7	11	0.71(0.51–0.98)	First/Second line
Rimawi et al. (PERTAIN) [12]	Trastuzumab+AI+/−Pertuzumab	251	18.9	15.8	0.65(0.48–0.89)	First line
Rimawi et al. (PERTAIN) [12] without induction chemotherapy	Trastuzumab+AI+/−Pertuzumab	110	21.7	12.4	0.55(0.34–0.88)	First line

*ET* Endocrine therapy, *MBC* Metastatic breast cancer

The recently published PERTAIN trial (with dual HER2-blockade therapy for MBC) showed the superior PFS benefit of pertuzumab plus trastuzumab and AI over trastuzumab and AI, especially for patients without chemotherapy, who reached a median PFS of 21.72 months [12]. The PERTAIN trial provides evidence that omission of chemotherapy may achieve comparable efficacy for certain low-risk subgroups with MBC. Additionally, the incidence of grade 3 adverse effects (AEs) in this subgroup of patients dramatically decreased from 66.2 to 28.3%, and AEs related to discontinuation of pertuzumab also declined from 33.8 to 17.0%. Therefore, ET with dual anti-HER2 treatments might offer an alternative effective and safe chemotherapy-sparing treatment regimen.

Additionally, with the advance of a de-escalating strategy in MBC management, it is important to find an alternative treatment with lower toxicity and comparable efficacy to the current standard first-line HER2+ MBC regimen (chemotherapy + trastuzumab + pertuzumab) in low-risk patients with less aggressive tumors. Further, from an economic perspective, since pertuzumab is not covered by insurance for metastatic disease and T-DM1 is currently unavailable in China, it is imperative to find alternative regimens with low-cost anti-HER2 agents that can help to reduce the socioeconomic burden of treatment strategies. Therefore, pyrotinib, a novel anti-HER2 tyrosine kinase inhibitor (TKI), exhibits great potential for first-line HER2+ MBC management.

Pyrotinib is a novel orally administered irreversible dual pan-ErbB TKI for HER2+ MBC. A phase II trial (NCT02422199) proved that pyrotinib plus capecitabine significantly prolonged median PFS versus lapatinib plus capecitabine (18.1 vs. 7.0 months; HR 0.363; 95% CI 0.228–0.579; *p* < 0.0001) [16]. This trial supported the hypothesis that pyrotinib may serve as a more potential anti-HER2 agent in the metastatic setting. Theoretically, dual anti-HER2 blockade with trastuzumab and pyrotinib provides a promising combination due to their different mechanisms of action and non-overlapping AEs profiles. The efficacy of this regimen has not yet been validated in previous studies.

In summary, previous studies have shown the significant benefit of ET plus dual anti-HER2 treatment, but this effect has not been validated for dual HER2 blockade with pyrotinib. Furthermore, it is currently unknown whether low-risk MBC patients would be suitable for a de-escalating treatment strategy without chemotherapy in the first-line setting. The aim of this trial is to evaluate the benefit of adding pyrotinib to ET and trastusumab as a standard treatment for a low-risk patient subgroup. The study is a randomized, open-label, phase II study, comparing the efficacy and safety of trastuzumab plus AI with or without pyrotinib for HR+/HER2+ MBC or inoperable locally advanced breast cancer (LABC) patients. Tumor samples obtained from pretreatment tumor and blood samples are to be collected for exploratory biomarker analyses to find predictive biomarkers and explore underlying resistance mechanisms.

## Methods

**Fig. 1:**
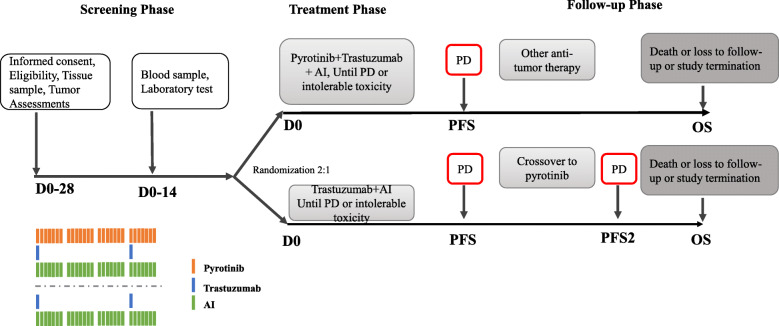
Schematic overview of study design

The present study is a multicenter, randomized, open-label, phase II study in HR+/HER2- metastatic breast cancer patients who have not received any prior systemic anti-cancer therapy for advanced disease (first-line setting). Eligible patients will be randomized in a 2:1 ratio to either of these groups: pyrotinib+ trastuzumab + an AI (Group A) or trastuzumab + an AI (Group B). The schematic overview of the study design is shown in Fig. 1.

### Study objectives

The primary objective of the study is to evaluate whether the combination of pyrotinib, trastuzumab and AI (Group A) will be superior to trastusumab plus AI (Group B). The primary endpoint is PFS, defined as the time from randomization to the first radiographically documented progression of disease or death from any cause, whichever will occur first. Disease progression will be evaluated according to the Response Evaluation Criteria In Solid Tumors Version 1.1 (RECIST 1.1) [17]. The secondary endpoints are overall survival (OS), objective response rate (ORR), duration of response (DoR), time to response (TTR), clinical benefit response (CBR), quality of life (QoL), safety, and tolerability. The exploratory endpoint is second progression-free survival (PFS2), defined as the time from randomization to second progression or death from any cause. PFS2 will be only recorded in crossover patients. Additional biomarker analyses are planned and regarded as exploratory analyses. Patients will be required to provide additional informed consent for those procedures. This study does not hypothesize about biomarkers or include any adjustment for type I errors. The aim is to highlight biomarkers or combinations of markers that may be predictive for efficacy or AEs.

### Inclusion criteria

To be eligible for inclusion in the trial, patients must provide written informed consent before the commencement of any study-related procedures. All patients must be above 18 years old with an Eastern Cooperative Oncology Group performance status 0–2 and a life expectancy of not less than 12 weeks. Eligible patients should be postmenopausal (fulfilling one or more National Comprehensive Cancer Network guideline criteria) or pre-menopausal with ovarian ablation or suppression. Furthermore, all participants should have pathological confirmed HR+/HER2+ metastatic or inoperable LABC with at least one measurable lesion evaluable according to RECIST 1.1. Also eligible are patients with asymptomatic brain metastases or stable brain metastases after local therapy (provided there is no clinical indication for immediate local re-treatment, as judged by the investigators).

Patients should have adequate bone marrow and organ function as defined by the following laboratory values at screening: neutrophil count ≥1.5 × 10^9^/L; platelet count ≥90 × 10^9^/L; hemoglobin ≥90 g/L; total bilirubin ≤1.5 × upper limit of normal (ULN); alanine transaminase (ALT) and aspartate transaminase (AST) ≤ 2 × ULN (ALT and AST ≤ 5 × ULN for liver metastases); creatinine ≤1.5 × ULN; left ventricular ejection fraction (LVEF) ≥ 50%.

### Exclusion criteria

The exclusion criteria are listed as follows: previous systemic non-hormonal anti-cancer therapy in the metastatic or advanced breast cancer setting; previous treatment with pertuzumab or T-DM1 in neoadjuvant or adjuvant treatment; extensive symptomatic visceral disease, severe organ dysfunction or disease considered by the investigator to be rapidly progressing or life-threatening, with a clinical indication for chemotherapy; the patient received the same AI as advised by the investigator within 7 days before randomization; uncontrolled central nervous system symptoms; disease-free interval from completion of adjuvant/neoadjuvant systemic non-hormonal treatment to recurrence less than 6 months; other malignancies within the last 3 years, except for carcinoma in situ of the cervix or basal cell carcinoma; major surgical procedure or significant traumatic injury within 28 days prior to the study treatment or anticipation of major surgery during the study period; inability to swallow or other factors that affect treatment administration; known hypersensitivity to any of the study drugs; a history of chronic heart failure of any New York Heart Association criteria, or serious cardiac arrhythmia requiring treatment (with the exception of atrial fibrillation and paroxysmal supraventricular tachycardia); a history of myocardial infarction within 6 months of randomization; a history of LVEF reduction to below 50% during or after prior trastuzumab neo-adjuvant or adjuvant therapy; pregnant or lactating women; QT interval > 470 ms; serious concomitant diseases (including severe hypertension, severe diabetes, active infection, thyroid disease, etc.) that are harmful to the patient’s safety or affect the patient’s completion of the study; the patient is assessed by the investigators to be unable or unwilling to comply with the requirements of the protocol.

### Interventions

Eligible patients will be randomized in 2:1 ratio to either Group A or Group B. A stratified randomization will be used to control confounding variables and balance the baseline characteristics between the different treatments. Patients will be stratified by the time since prior ET (< 12 months/≥ 12 months/no prior ET) and location of metastatic lesions (visceral versus non-visceral).

Patients randomized to Group A will receive pyrotinib + trastuzumab + AI and patients randomized to Group B will receive trastuzumab + AI. In each group, trastuzumab will be administered every 3 weeks intravenously (8 mg/kg loading doses followed by 6 mg/kg maintenance doses). Pyrotinib will be administered 400 mg orally daily. For ET, all pre- and perimenopausal women will receive ovarian ablation or suppression. The AI is to be determined by investigators before randomization. The available options for AI could be either anastrozole, letrozole, or exemestane (1 mg/2.5 mg/25 mg, once daily, oral). Patients will continue to receive the assigned medication until objective disease progression, symptomatic deterioration, or unacceptable toxicity (at the discretion of the treating physician), death or withdrawal of consent, whichever occurs first. The imaging evaluation will be performed according to the RECIST 1.1. The patients in Group B will be eligible for crossover to Group A in case of disease progression.

Patients will be screened at baseline for eligibility. And providing signing informed consent, baseline measurements will be obtained. Survival status will be followed every 12 weeks regardless of treatment discontinuation until death, lost to follow-up, or withdrawal of consent to survival follow-up. Survival information can be obtained via phone, and information will be documented in the relevant case report form. Any post-study cancer treatment will be recorded. All patients will be followed for safety up to 28 days after the last dose of study treatment (pyrotinib/trastuzumab/AIs). For patients who discontinue due to reasons other than progressive disease (PD), death, lost to follow-up or withdrawal of consent to efficacy follow-up, tumor assessments and patient-reported outcomes must continue to be acquired every 6 weeks until PD, death, lost to follow-up, or withdrawal of consent to efficacy follow-up. Group B patients who choose to cross over after first objective progression will have their progression status recorded every 6 weeks to assess time to the second PD.

Schedule of enrollment, interventions, and assessments are shown in the supplementary material.

### Statistics: sample size and power calculation

The primary objective of this study is to compare the PFS between the two groups. The hypothesis is based on hazard ratio (HR): H0: HR = 1; Ha: HR ≠ 1. In the case of 2:1 allocation, a sample of 180 evaluable patients with 144 events is expected to provide 80% power (significance level 0.05) to detect an improvement in median PFS, from 8.0 months with trastuzumab plus an AI to 13.0 months when adding pyrotinib [PASS 15]. Taking the withdrawal rate as approximately 10%, the sample size will be 198 patients.

### Statistics: analyses

#### Efficacy analyses

The difference in PFS (primary endpoint) will be estimated using the full analysis set (FAS) in a stratified log-rank test accounting for all stratification factors and a Cox proportional hazards model to explore baseline factors that may affect PFS. Secondary objectives include comparisons of OS, ORR, TTR, DoR, CBR, QoL, and safety. A stratified log-rank test (using the same stratification factors as for the PFS analysis) will be used to compare OS, TTR and DoR between the two groups. Kaplan-Meier curves, median PFS/OS, TTR, and DoR HR with appropriate confidence intervals will be reported. Exploratory analyses will be conducted for PFS2.

A 95% CI for ORR/CBR will be provided. The comparison for response rates between the two groups will be assessed using the Cochran-Mantel-Haenszel test with the same stratification factors as for the PFS analyses. Analyses of ORR/CBR will be performed on the FAS population.

#### Safety analyses

The type, grade and frequency of AEs will be recorded. AEs and abnormal findings in laboratory tests will be listed with the relationship to the study treatments. The AE summary tables for crossover patients will include all AEs that occurred after the start of crossover treatment until the end of the 30-day follow-up period.

## Discussion

This study aims to verify that the addition of pyrotinib to trastuzumab plus AI may convey survival benefits to HR+/HER2+ MBC or LABC patients. Since there is a scarcity of existing trials on AI with dual HER2 blockade, this study aims to generate evidence that pyrotinib combined with trastusumab and AI could be used as a de-escalating treatment with less toxicity and comparable efficacy for patients.

As a substantial number of patients in the ALTERNATIVE [11] and PERTAIN [12] trials received chemotherapy, the effect of AI and anti-HER2 agents is likely to have been masked in these two trials. Subgroup analysis of the PERTAIN trial indicated that patients who refused the induction chemotherapy showed significant survival benefit with an additional anti-HER2 agent, while patients who had chemotherapy did not benefit from dual anti-HER2 blockage. Additionally, chemotherapy may have severe AE outcomes, which have the potential to compromise therapeutic compliance. Therefore, it is important to further evaluate the effect of ET as an alternative to chemotherapy for HER2+ MBC in order to deliver efficacious treatments with a favorable safety profile. The present study was designed to conduct a thorough comparison in low-risk patients without the administration of chemotherapy to validate the efficacy of dual HER2 blockade with endocrine therapy. If the present study complies with this hypothesis, phase III trials would focus on the following issues: 1) whether AI + trastuzumab + pyrotinib is superior to AI + trastuzumab + pertuzumab; 2) high-risk patients could be included and treated with chemotherapy plus dual-HER2 blockade as induction therapy. If the participants respond well, further studies would evaluate whether AI plus trastuzumab and pyrotinib would be effective as maintenance treatment.

The reason for excluding patients with previous exposure to pertuzumab and T-DM1 is that patients pre-treated by pertuzumab and T-DM1 experienced relapse, which indicated that the cancer was likely to have had an aggressive biological behavior and more intensive treatment may be needed. Therefore, this subgroup of patients may not be suitable for this de-escalating strategy with only ET and anti-HER2 therapy. Furthermore, since T-DM1 is not available in China and pertuzumab was only obtainable last year and was covered by insurance for the neoadjuvant and adjuvant setting only, it could be speculated that in the accrual period of the present project, patients who were previously exposed to pertuzumab and T-DM1 may only account for a very small proportion of patients with HER2+ MBC. Including this patient subgroup may potentially increase the heterogeneity of participants and undermine the strength of the final conclusion. Therefore, the study design excluded patients that were previously treated with pertuzumab or T-DM1 and defined a low-risk MBC subgroup that may benefit from AI and anti-HER2 therapy with the omission of chemotherapy. The study design could also potentially increase the homogeneity of study participants, which could provide more robust evidence for future studies.

In addition, prespecified biomarker and subgroup analyses are also included in this protocol. These may extend current knowledge on the prognostic and predictive value of available clinicopathological parameters and may also help to define a suitable MBC patient subgroup with potential survival benefit with the addition of pyrotinib. Of note: the present study focuses on a low-risk patient subgroup to implement a de-escalating strategy for HER2+ MBC. The generalization of results to patients with heavy disease burden or rapidly progressive disease should be made with great caution.

## Supplementary information

**Additional file 1.**

## Data Availability

Not applicable.

## Abbreviations

AEs: Adverse Effects
AI: Aromatase Inhibitors
CBR: Clinical Benefit Response
DoR: Duration of Response
FAS: full analysis set
ER: Estrogen Receptor
ET: Endocrine Therapy
HER2: Human Epidermal Growth Factor Receptor 2
HR: Hormone Receptor
LABC: Locally Advanced Breast Cancer
LVEF: Left Ventricular Ejection Fraction
MBC: Metastatic Breast Cancer
ORR: Objective Response Rate
OS: Overall Survival
PD: Progressive Disease
PFS: Progression-Free Survival
PFS2: Second Progression-Free Survival
QoL: Quality of Life
RECIST: Response Evaluation Criteria in Solid Tumors
TKI: Tyrosine Kinase Inhibitor
TTR: Time to Response
ULN: Upper Limits of Normal
