# Supplementary material for: Pyrotinib with trastuzumab and aromatase inhibitors as first-line treatment for HER2 positive and hormone receptor positive metastatic or locally advanced breast cancer: study protocol of a randomized controlled trial
Source: BMC Cancer. 2020 Jul 13;20:653. doi: 10.1186/s12885-020-07143-2 (PMC7359576; doi:10.1186/s12885-020-07143-2)
Supplement: Supplementary file 1 — Additional file 1. [file 12885_2020_7143_MOESM1_ESM.docx]

# Overview

This is a prospective, randomized, open label study. 198 patients with HER2+/hormone receptor (HR)+ metastatic breast cancer (MBC) will be recruited. Eligible patients will be allocated (2:1) to either of these groups: an experimental group (pyrotinib + trastuzumab + aromatase inhibitors [AI]) or a control group (trastuzumab + AI). Allocation will be stratified by 1) time since adjuvant hormone therapy (≤ 12 months/> 12 months/no prior hormone therapy); 2) lesion sites (visceral; non-visceral). The primary endpoint is progression-free survival (PFS).

After enrollment, the subjects will receive continuous administration of the treatment, with 21 days as one cycle, until disease progression, intolerable toxicity, withdrawal of consent or requirement of drug discontinuation, as judged by the investigator.

After treatment, safety issues will be followed up continuously until 28 days after the last dose, all adverse events are ≤ grade 1, or clinical stability of all adverse events (whichever comes later). The subjects who are excluded for reasons other than progressive disease (PD) or death are to be followed for efficacy evaluations until PD, receipt of other antitumor therapies or death (whichever comes first). Survival data will be assessed (collection of overall survival [OS] data) for all subjects, until death or the end of OS data collection (whichever comes first). An electronic data collection (EDC) system is to be used for collection of clinical data in this study. No interim analysis is planned for the present study.

# Schedule of enrollment, interventions and assessments

| Time point | Study period | | | |
| --- | --- | --- | --- | --- |
|  | Screening Phase | Treatment Phase | Follow-Up Phase | |
|  | Baseline | Treatment | Treatment Discontinuation | Follow-Up |
| Eligibility | X |  |  |  |
| Informed consent | X |  |  |  |
| Quality of life (QoL) | X | X | X |  |
| ECOG performance status | X | X | X |  |
| Complete physical examination | X | If clinically indicated | X |  |
| Vital signs | X | X | X |  |
| Symptom-directed physical exam | X | X | X |  |
| Tumor tissue for pathological diagnosis | X |  |  |  |
| 12-lead electrocardiogram (ECG) | X | X | X |  |
| Chest X-ray |  | If clinically indicated |  |  |
| LVEF by ECHO | X | X | X |  |
| Tumor assessments | X | X | X |  |
| Treatment |  | X |  |  |
| Hematology and blood chemistry | X | X | X |  |
| Pregnancy test | X |  | X |  |
| INR and aPTT | X | X | X |  |
| Serum and plasma for biomarker analysis | X | X | X |  |
| Tumor tissue for biomarker analyses | X |  |  |  |
| Estradiol | X | X |  |  |
| Bone scan |  | If clinically indicated |  |  |
| Concomitant medications and cancer-related surgery/procedures | X | X | X | X |
| Adverse events | X | X | X |  |
| Survival information |  |  |  | X |

aPTT, activated thromboplastin time; INR, international normalized ratio

# Dose modifications

At the discretion of the investigator, pyrotinib may be delayed if necessary. Resumption of treatment with pyrotinib is allowed after a delay. Pyrotinib dosage should start with the next lower level after resumption (dose level 400/320/240 mg, with a minimum of 240 mg).

During treatment, multiple delays or adjustment of pyrotinib are permitted, and treatment should continue after the adverse event returns to Grade 0-1 and the complications disappear. The cumulative duration of pause in each cycle should not exceed 14 days to ensure drug efficacy. Patients will cease treatment if the duration of pyrotinib does not meet the above criteria.

There is no need to take extra doses to make up the missed doses.

Table: Dose adjustment for pyrotinib

| NCI CTCAE 4.0* | Dose adjustment | Dose after recovery |
| --- | --- | --- |
| Cardiotoxicity | | |
| ≥ 20% drop in LVEF /  LVEF ≤50% | Permanently discontinued | - |
| Diarrhea | | |
| Grade Ⅳ | Permanently discontinued | - |
| Grade Ⅲ | Delay, until the recovery to Grade 0-1, and complications have disappeared | First dose：400 mg  Second dose：320 mg |
| Grade I~II with complications  (including but not limited to ≥ grade II nausea or vomiting, fever, bleeding or dehydration) |  |  |
| Other AE | | |
| ≥ Grade II non-hematologic adverse events (except for hair loss, fatigue, fatigue, etc.) | Delay, until recovery to Grade 0-1, and complications have disappeared | First dose：400 mg  Second dose：320 mg |
| ≥ Grade III hematologic adverse events | Delay, until recovery to Grade 0-1, and complications have disappeared | First dose：400 mg  Second dose：320 mg |

CTCAE, Common Terminology Criteria for Adverse Events; LVEF, left ventricular ejection fraction. NCI, National Cancer Institute. Clinical treatment or observation by the investigator are based on the patients and adverse events. If adverse events continue to appear (< 14 days), it is recommended that the medication should be adjusted according to this form.

Trastuzumab can be stopped or delayed to assess or treat adverse effects (AE). No dose reductions are allowed. The detailed regimen will follow the local label and guidelines. Delays, reductions or discontinuations of AI are based on the label and local guidelines in China.

# Data analysis sets

The analysis set in this study includes full analysis set (FAS), per protocol set (PPS) and safety set.

FAS is an analysis set determined in accordance with the intention-to-treat principle. All the subjects who are randomized and have received at least one dose of investigational product will be included in this set. The FAS is the primary analysis set for efficacy analysis in this study.

PPS is a subset of FAS. All the subjects who are randomized, have received at least one dose of investigational product and have no important deviation from the protocol will be included in this analysis set.

Safety set: all the subjects who are randomized and have received at least one dose of investigational product will be included in the analysis set.

# Permitted or prohibited during the trial

Patients can receive best supportive care. Active treatment should be administered if there are clinical concomitant diseases and various AEs.

During the treatment period, anti-tumor drugs and adjuvant drugs related to breast cancer therapy should be discontinued, including anti-tumor traditional Chinese medicine and other immunological preparations.

# Data collection

An electronic case report form (eCRF) is used for data collection in this study; Jiangsu Hengrui Medicine Co., Ltd. will provide study institutions with the EDC system.

The nurses or clinical research coordinators will use the EDC system for the designated personnel at the study institutions. The study staff at the study institutions cannot log into the EDC system until their training is complete. Investigators or dedicated data entry operators should enter data into the EDC system in accordance with the visit schedule requirement and the eCRF completion guideline.

# Protocol violation

All the requirements specified in the protocol must be followed strictly. Any intentional or unintentional behavior of deviation from or violation of the trial protocol could be classified as a deviation from or a violation to the protocol. Investigators should document the protocol violation and record the time of the finding, the onset time, the event process, the reasons for the violation and any corresponding measures undertaken if there is found to be any deviation from the protocol. The record should be signed by the investigators and reported to the ethics committee.

# Data management

The investigators should agree to maintain all study materials, including the original record during hospitalization, informed consent, the case report form and the detailed record of drug dispensation.

The study materials should be maintained by the research institution until 5 years after the end of the clinical trial.

# Data imputation

Efficacy parameters: all the missing values in the main efficacy parameters due to premature withdrawal will be regarded as “unable to be evaluated”. For calculation of the time-to-event variables involved (e.g. PFS and OS), subjects whose post-treatment radiological evaluation is missing will be checked case by case to determine their censored times.

# Serious adverse event (SAE)

A serious adverse event (SAE) is defined as a medical event requiring hospitalization, prolonged hospital stay, disability, affects working ability, is life-threatening or fatal, or leads to congenital malformation during the clinical trial. The unexpected medical events are included below:

1). Events that result in death;

2). Life-threatening events (defined as risk of death at the onset of the event);

3). Events that require in-patient hospitalization or prolonged hospitalization;

4). Events that resulted in persistent or significant disability/incapacity; a congenital abnormality or birth defect;

The occurrence, clinical control and prognosis of all serious adverse events should be followed up by investigators until recovery to normal, relief or stability, and recorded in the original medical record, the eCRF in detail (including the adverse event report form; the death report form must be filled in in case of death record), and a SAE report form. Serious adverse events should be monitored for 28 days (inclusive) from the signing of the informed consent form until the last dose of study drug. SAEs during the trial should be reported within 24 hours of discovery, and in case of death must be reported immediately. All deaths should be reported to the ethics committee of the study unit, the general office of the China Food and Drug Administration and the local (provincial or municipal) Food and Drug Administration where the investigators are located. All SAEs that occur 28 days after the last dose do not need to be reported unless they are related to the study drug.

The record of the SAE should include detailed symptoms, severity, time of onset, time of management, measures taken, follow-up time and method, as well as outcome. If the investigators consider that a SAE is not related to the study drug but is potentially related to the study condition (e.g., termination of the original treatment or comorbidity during the trial), this should be described on the SAE page of the case report form. All SAEs must be followed up until the patient has recovered or is stable.

# Protocol amendments

Except for the principal investigator, no one can make amendments to the protocol. Any change to the protocol must be made on an amendment form, and submitted to the ethics committee for approval or the record kept after signature by the principal investigator. Detailed information on historic amendments should be described in the protocol.

# Consent or assent

Subjects must be informed of participation in the trial and provide written approval prior to the start of study treatment to ensure their legal rights. Investigators are responsible for a complete and full introduction of the objectives of this study, drug effects, possible side effects and risks to subjects, and should also clarify patients’ rights, risks and benefits. Conversation is a very important process of informed consent. If the subject and her legal representative have diminished cognitive ability, a witness should be involved in the informed consent process. In such a case, oral consent, the signature of the subject/her legal representative, together with the signature of the witness are required on the same day. The version number and date should be noted on the informed consent form.
